# Exploring Pregnancy-Related Information-Sharing Behavior Among First-Time Southeast Asian Fathers: Qualitative Semistructured Interview Study

**DOI:** 10.2196/58482

**Published:** 2024-12-09

**Authors:** Kidung Ageng, Anushia Inthiran

**Affiliations:** 1 Department of Accounting and Information Systems University of Canterbury Christchurch New Zealand

**Keywords:** pregnancy, first-time fathers, information sharing, Southeast Asia, information-seeking behavior, cultural factors

## Abstract

**Background:**

While the benefits of fathers’ engagement in pregnancy are well researched, little is known about first-time expectant fathers’ information-seeking practices in Southeast Asia regarding pregnancy. In addition, there is a notable gap in understanding their information-sharing behaviors during the pregnancy journey. This information is important, as cultural norms are prevalent in Southeast Asia, and this might influence their information-sharing behavior, particularly about pregnancy.

**Objective:**

This study aims to explore and analyze the pregnancy-related information-sharing behavior of first-time expectant fathers in Southeast Asia. This study specifically aims to investigate whether first-time fathers share pregnancy information, with whom they share it, through what means, and the reasons behind the decisions to share the information or not.

**Methods:**

We conducted semistructured interviews with first-time Southeast Asian fathers in Indonesia, a sample country in the Southeast Asian region. We analyzed the data using quantitative descriptive analysis and qualitative content theme analysis. A total of 40 first-time expectant fathers were interviewed.

**Results:**

The results revealed that 90% (36/40) of the participants shared pregnancy-related information with others. However, within this group, more than half (22/40, 55%) of the participants shared the information exclusively with their partners. Only a small proportion, 10% (4/40), did not share any information at all. Among those who did share, the most popular approach was face-to-face communication (36/40, 90%), followed by online messaging apps (26/40, 65%). The most popular reason for sharing was to validate information (14/40, 35%), while the most frequent reason for not sharing with anyone beyond their partner was because of the preference for asking for information rather than sharing (12/40, 30%).

**Conclusions:**

This study provides valuable insights into the pregnancy-related information-sharing behaviors of first-time fathers in Southeast Asia. It enhances our understanding of how first-time fathers share pregnancy-related information and how local cultural norms and traditions influence these practices. In contrast to first-time fathers in high-income countries, the information-sharing behavior of first-time Southeast Asian fathers is defined by cultural nuances. Culture plays a crucial role in their daily decision-making processes. Therefore, this emphasizes the importance of cultural considerations in future discussions and the development of intervention programs related to pregnancy for first-time Southeast Asian fathers. In addition, this study sheds light on the interaction processes that first-time fathers engage in with others, highlighting areas where intervention programs may be necessary to improve their involvement during pregnancy. For example, first-time fathers actively exchange new information found with their partners; therefore, creating features or platforms that facilitate this process could improve their overall experience. Furthermore, health practitioners should take a more proactive approach in engaging with first-time fathers, as currently there is a communication gap between them.

## Introduction

### Background

In the realm of pregnancy, the traditional focus has predominantly centered on mothers as the primary seekers and bearers of knowledge [1]. However, contemporary perspectives increasingly emphasize the integral role of fathers in the journey to parenthood [2]. Fathers’ participation in maternal-fetal health care offers profound benefits; for example, it is associated with a reduction in birth-related risks [3] and improved cognitive outcomes in infants [4]. Consequently, the Cairo Initiative [5] and recommendations from the World Health Organization [6] advocate for the promotion of a father’s engagement during pregnancy. The Cairo Initiative emphasized the importance of reproductive health and the shared responsibilities of men and women in family planning and health-related decisions while promoting men’s involvement in pregnancy to support maternal health and ensure better pregnancy outcomes.

Fathers have emerged as active participants in the pursuit of pregnancy-related information [7,8]. This is one of the many ways fathers become involved in the pregnancy journey, which involves obtaining information about pregnancy to improve their knowledge [2]. Fathers navigate the vast landscape of the internet and engage with specialized app, websites, blogs, and online support groups [9-12]. This digital terrain resonates particularly with millennial fathers, who readily turn to internet-based resources for guidance and support [13]. While fathers’ information-seeking practices have garnered significant attention, it is crucial to acknowledge that the dynamics of this journey transcend mere information acquisition and extend to the pivotal aspect of information sharing. This is because fathers also actively engage with others to exchange information, experiences, insights, and support throughout the pregnancy journey [14].

Information sharing is a multifaceted aspect of human communication, driven by various motivations and yielding both advantages and disadvantages. People engage in sharing to disseminate knowledge, experiences, and insights, fostering collaborative and interactive communication [15,16]. The process is influenced by key variables, such as trust, risk assessment, perceived rewards, and the dynamics of relationships [17,18]. A rapid review study by Naeem et al [18] also emphasizes privacy concerns and the sensitivity of information as the multifactorial nature of an individual’s willingness to share health information. Trust plays a pivotal role, as individuals are more inclined to share sensitive information when there is trust in the audience [18]. Assessing the risk associated with sharing and anticipating potential rewards, such as support or advice, further motivates information-sharing behaviors [15,17]. The advantages of sharing include the creation of a supportive community, the exchange of diverse perspectives, and the building of relationships [19]. Sharing can enhance one’s understanding, contribute to a collective pool of knowledge, and provide emotional and practical support [10,19]. However, sharing also comes with disadvantages. Individuals may face the risk of judgment or misunderstanding [20] and the potential harm of information misuse or misinterpretation [21]. Striking a balance between the benefits and drawbacks of sharing is a nuanced consideration in various contexts, influencing individuals’ decisions to engage in information-sharing behaviors.

In the domain of pregnancy information sharing, results of a research study demonstrate that fathers exhibit a willingness to share information acquired about pregnancy and fatherhood [11]. Fathers actively engage in both online and offline practices [22]. In the context of online information sharing, fathers contribute to discussions and seek advice within their digital communities [11]. They share relevant articles or seek guidance on parenting forums [10]. Some fathers disseminate their knowledge through online forums, while others opt to articulate their experiences via blogs [10]. Within these online forums, fathers recount their journey and provide mutual support [23]. In addition, fathers frequently indicate their participation in online support groups tailored to discussing various aspects of the pregnancy and fatherhood experience [24]. Furthermore, fathers have embraced social media apps as platforms for expressing their emotions and recounting their pregnancy journeys [9]. In these internet-based spaces, fathers actively share pregnancy-related information, fostering their involvement, bolstering their confidence, and encouraging their peers [25,26]. Conversely, offline information sharing predominantly takes place through face-to-face conversations. This emphasizes the significance of interpersonal relationships in the dissemination of information [27]. Fathers engage in discussions about pregnancy-related experiences during casual hangouts or directly seek advice from family members and friends [28]. This practice is driven by the desire to connect, collaborate, and contribute to collective knowledge [19].

While certain fathers actively share information about pregnancy with others [9,11,23], results of other studies suggest that some fathers may refrain from sharing pregnancy-related information [20,29]. First-time fathers, in particular, may seldom share personal experiences about the pregnancy journey, often due to feelings of unfamiliarity with the topic [29]. In addition, they may choose to keep pregnancy matters confidential, adhering to local cultural norms that discourage fathers from discussing such matters with others [29]. As seen in a previous study by Esmaeilzadeh [30], privacy concerns often influence the extent to which individuals are willing to share sensitive information, particularly in online environments like social media. A study by Nash [20] also reveals that, even though fathers share health information about pregnancy, they may withhold their feelings related to pregnancy or fatherhood due to concerns about judgment from other men, indicating a thoughtful risk assessment before sharing the information. These findings illuminate various reasons why fathers may opt not to share information, thereby limiting their potential benefits from engaging in this practice [31].

Studies on fathers’ information-sharing behavior in the context of pregnancy information in high-income countries are well-researched [11,23,26]. However, there is a noticeable gap in research studies addressing pregnancy-related information-sharing practices in low- and middle-income countries, particularly in the Southeast Asian context. Southeast Asia is characterized by its diverse cultural norms, traditions, and varying degrees of digital literacy [32-34], providing a unique backdrop for examining how fathers participate in the sharing of pregnancy-related information. In the Southeast Asian context, cultural norms of privacy and reserved communications may impact fathers’ information-sharing behaviors [30], influencing whether sharing is performed, what information fathers share, and with whom they share pregnancy-related information [35]. The cultural emphasis on societal expectations within Southeast Asian cultures further directs fathers toward traditional channels, such as consulting with extended family members or seeking advice from older people [36,37].

Adding to this landscape, Southeast Asia exhibits varying levels of digital proficiency, impacting how fathers share information and engage in online information sharing [38]. Specifically, this study was conducted in Indonesia, the most populous nation in Southeast Asia. Indonesia, being part of Southeast Asia, embodies a rich tapestry of diverse cultural norms, traditions, and varying levels of health and digital literacy [38-40]. The country’s internal diversity provides a nuanced backdrop for exploring how first-time expectant fathers engage in sharing pregnancy-related information. Indonesia’s cultural, linguistic, and contextual elements mirror the broader diversity within Southeast Asia [34,41,42]. The shared cultural threads and regional commonalities offer insights that may extend to other Southeast Asian countries [39,43].

Southeast Asia is a region with various ethnicities, religions, and languages [34]. These differences may impact the information-sharing behaviors of first-time expectant fathers. For example, fathers from Malay, Chinese, or Indigenous communities may have distinct traditions and customs related to pregnancy [36]. These differences may influence their communication patterns and the type of information they prioritize to search and share (or not to share). In addition, religious practices related to Islam, Christianity, Buddhism, and Hinduism may guide the behavior and decision-making process of fathers during pregnancy [44,45]. Finally, linguistic diversity may shape how fathers search for and share pregnancy-related information using their preferred language. Results of a research exploring cross-language information-seeking behavior showed participants implement different strategies and tactics when completing the search process depending on the language they used [44]. Therefore, fathers might have different strategies and approaches when searching and sharing, depending on the language used during the process.

### This Study

An exploration of information-sharing practices in these contexts contributes valuable insights to our understanding of how these nuances shape fathers’ behavior in the pregnancy journey, as it is likely to be different from the results of research studies conducted in high-income countries. Therefore, this study embarks on an exploration of information-sharing practices of pregnancy information among first-time fathers in Indonesia, a Southeast Asian low- and middle-income country, where cultural traditions and social networks intersect and may shape fathers’ behaviors [45]. Our research question and follow-up questions are outlined as follows: Do first-time expectant fathers engage in information sharing about pregnancy? The follow up questions were as follows: (1) If information is shared, with whom is it shared? (2) Through what means the information is shared? (3) Why is the information shared? and (4) If not shared, what are the underlying reasons? This exploration is particularly salient within the temporal confines of the antenatal phase, a pivotal juncture in the transition to fatherhood [46]. Here, we draw a clear distinction between first-time fathers and those with prior parenting experience. The rationale is that first-time fathers bring a unique perspective as they prepare for fatherhood [29]. Furthermore, first-time fathers display heightened sensitivity to their new situation, leading to more pronounced information needs and interaction compared to fathers with prior experience [47]. The results of this study aim to enhance domain knowledge by providing answers to the research questions stipulated earlier.

## Methods

### Overview

In this study, a grounded theory approach [48] was used to investigate the information-sharing practices of first-time expectant fathers concerning pregnancy-related information. Grounded theory enables the development of theories derived directly from empirical data and provides a neutral perspective on human actions within a social context [49]. The grounded theory approach was used for this study due to the limited knowledge regarding the information-sharing practices of first-time fathers in the context of pregnancy-related information in Southeast Asia. This methodology facilitates the discovery of patterns, capturing lived experiences, exploring the themes, and systematically developing theories grounded in the data itself [50]. This methodological choice is particularly valuable in the context of this study as it allows for the in-depth exploration of the nuanced and context-specific behaviors of first-time fathers beyond surface-level observations. To enhance interpretive validity and intercoder reliability, personal reflexivity practices were implemented [51].

### Recruitment

Recruitment notices were placed in health care facilities in Indonesia, community centers, and online social media portals. Participants were required to meet specific inclusion or exclusion criteria. Participants must be aged ≥21 years, first-time fathers, and have sought pregnancy-related information in the past 9 months. Importantly, participants must not be working in or have a formal qualification or be in the process of achieving an academic qualification in the health care or allied health care sectors to eliminate the possibility of health expertise and literacy influencing results.

### Data Collection

Data collection involved the use of questionnaires and semistructured interviews. The questionnaire serves to capture information on sociodemographic, digital and health literacy levels, family situations, and pregnancy-related details. This is to provide an overview of the participants of this study and will not be used for analysis. The sociodemographic section encompasses details about participants’ social and demographic characteristics. Digital literacy levels are self-assessed to gauge participants’ proficiency and comfort with digital technologies. Similarly, health literacy levels are self-assessed to understand participants’ capacity to comprehend health-related information effectively. These self-assessments provide insights into fathers’ perceived literacy levels related to health and digital matters. Family situations are explored to uncover contextual factors that may influence information-sharing behaviors, considering the role of familial connections in the pregnancy journey. Finally, pregnancy-related details are gathered to gain insights into specific aspects of participants’ experiences.

Semistructured interviews were used to collect data, enabling in-depth exploration of participants’ thoughts and experiences [52]. The interviews were designed to be open-ended and revolved around the research questions outlined in this study. Participants were queried about their engagement in the sharing or nonsharing of pregnancy-related information. Participants who shared information were asked follow-up questions: (1) with whom the information is shared, (2) through what means the information is shared, and (3) why the information is shared. Participants who did not share pregnancy-related information were asked to elaborate on the reasons for this decision. Participants were allowed to choose whether the interview will be conducted in Bahasa Indonesia or English. An option was provided, as using the mother tongue in interviews can lead to more reliable and valid data [53]. The researcher is Indonesian, so the interview and translation were conducted by the researcher if the participants chose the interview to be conducted in Bahasa Indonesia. If the interview was conducted in Bahasa Indonesia, transcribing was done in Bahasa Indonesia to prevent the loss of meaning of the answers and collect as much culturally localized information as possible [54]. Thereafter, the interviews were translated into the English language [53]. All interviews were recorded and transcribed verbatim, ensuring a robust connection between the data and the researcher [55].

### Data Analysis

Open coding technique was used with the grounded theory approach [56]. This method was used to examine participants’ responses regarding their information-sharing practices [57]. Initially, a master list of codes was generated through inductive reasoning, with ongoing revisions after every third interview [58]. Subsequently, these codes were condensed into overarching themes using the constant comparative method, which entailed a systematic review of each response to identify prominent patterns and key themes [58,59]. Data analysis commenced immediately following each interview and continued until the study’s completion [50]. This method of content analysis provided a comprehensive understanding of fathers’ information-sharing practices about pregnancy and the underlying reasons for their behavior [50]. To ensure interpretive validity and intercoder reliability, personal reflexivity practice was undertaken [51].

### Ethical Considerations

Before recruitment, ethics approval was obtained from the University of Canterbury, New Zealand (HREC 2022/66). Informed consent was obtained from all participants before their involvement in the study. Participants were provided with a detailed information sheet consisting of the study’s objectives, procedures, potential risks, and benefits. They were also informed of their right to withdraw from the study at any time without any penalty. Their consent was recorded electronically. All identifiable information was anonymized, and unique codes were assigned to each participant. Participants were informed about how their data would be stored and used, which is based on the university’s regulations. Data were stored securely on password-protected devices to prevent unauthorized access. There was no compensation for participants.

## Results

### Overview

In this section, we begin with a brief overview of the sociodemographic details of the participants. Following this, we delve into participants’ information-sharing behaviors, categorized into those who shared information and those who did not. Information-sharing activities among first-time fathers who shared information are further explored, considering the audience, communication channels used for sharing information, and underlying reasons for sharing. In addition, further examination of first-time fathers who chose not to share information will be provided, revealing their underlying reasons behind this decision.

### Sociodemographic Details of Participants

There were 40 participants in this study. Data saturation [60] for this study was achieved with 75% (30/40) participants. The data saturation point is generally reached in 15 interviews, with one-third of the number of interviews being added [61]. Similarly, a study showed that thematic saturation from the conducted interviews will be achieved by completing 30 interviews [62]. Furthermore, other studies exploring information-seeking behavior recruited 40 participants for the interviews to reach data saturation [63,64]. Therefore, in this study, after data saturation was achieved with 30 participants, the researcher added another one-third of the number of interviews, bringing the total to 40 participants.

Most participants fell within the age group of 26 to 30 years, constituting 68% (27/40) of the sample. Urban residency was predominant, with the majority residing in cities (21/40, 52%), while others living in suburbs (10/40, 25%) and rural areas (9/40, 22%). Educational backgrounds were diverse, with 65% (26/40) holding bachelor's degrees, followed by master’s degrees (7/40, 18%), and high school qualifications (7/40, 18%). Language preferences for online searches indicated that most used the national language (Bahasa Indonesia; 25/40, 62%), rather than the local languages or dialects (ie, Javanese, Sundanese, Balinese, and Betawi). A national language is a language that is officially recognized and used formally at the country level. By contrast, local languages or dialects are specific to certain communities or regions with specific cultural markers. Most (24/40, 60%) participants worked in the private sector, followed by entrepreneurs (8/40, 20%). Monthly household income varied, with 45% (18/40) falling within the IDR 2-4.99 million (US $120-300) range, followed by the IDR 5-9.99 million (US $325-650) range (14/40, 35%).

In terms of social and familial aspects, 88% (35/40) identified with the Islamic faith. Furthermore, 62% (25/40) of participants perceived their family social systems as equal, including roles and responsibilities between husband and wife. Most (36/40, 90%) participants lived together with their partners. Most participants’ partners were in the third trimester (20/40, 50%), followed by the first and second trimesters equally (10/40, 25%). Health and digital literacy levels varied, with 60% (24/40) of participants having moderate health literacy and 28% (11/40) having high health literacy. In terms of digital literacy, 50% (20/40) demonstrated high digital literacy, while 40% (16/40) exhibited moderate digital literacy.

### Shared or Did Not Share

Results indicated that 90% (36/40) of the participants shared information, while 10% (4/40) of the participants did not share information with anyone. Within the 36 participants, 55% (22/40) participants only shared information with their partners. Furthermore, 20% (8/40) of the participants shared information solely with their partners, extended family, and the physicians. Results indicated that most (36/40, 90%) first-time fathers were “sharers” of pregnancy information.

### Information-Sharing Activities of First-Time Fathers

#### The Audiences: Who Is Information Shared With?

Table 1 details participants’ information-sharing patterns regarding pregnancy. Most (22/40, 55%) participants exclusively shared information with their partners. The second prevalent pattern involved a combination of sharing with partners, physicians, and extended family members (eg, mothers, siblings with pregnancy experience, or siblings with medical qualifications), amounting to 20% (8/40) of the participants. Furthermore, 10% (4/10) of the participants opted to share information with physicians, friends, colleagues, and their partners. Only a small proportion, 5% (2/40), extended their sharing to a social media audience, encompassing participants’ social media connections or followers, in addition to partners, physicians, and friends. The findings indicated that fathers primarily share information with their partners only.

**Table 1:** Information sharing behavior of fathers (N=40).

Activity and audience		Participants, n (%)
Did not share information		4 (10)
Shared information		36 (90)
**Shared information with**		
	Partner only	22 (55)
	Partner, physicians, and extended family	8 (20)
	Partner, physicians, friends, and colleagues	4 (10)
	Partner, physicians, friends, and social media audiences	2 (5)

#### The Channels: How Is Information Shared?

Participants shared information using offline and online communication channels. Face-to-face communication was the favorite offline method used, as 90% (36/40) opted to share information with this method. As for online communication channels, 65% (26/40) of the first-time fathers used online messaging apps such as WhatsApp (Meta Platforms, Inc) or iMessage (Apple Inc) to share information. With this method, first-time fathers not only shared information using the text format but also sent links and screen captures. Aside from online private messaging platforms, 35% (14/40) of the first-time fathers shared information found on online posts or videos using social media features available on Instagram (Meta Platforms, Inc) and Facebook (Meta Platforms, Inc), such as “direct sharing.” However, the “tagging” feature in social media platforms is not a favored method of sharing information, chosen by only 10% (4/40) of the fathers. In addition, only 5% (2/40) leveraged social media “post” features and “comment sections” to disseminate pregnancy information.

The results from Table 2 illustrate the diverse combination strategies used by first-time fathers in sharing pregnancy-related information. The participants in this study used various communication channels to share pregnancy information with their audiences. Face-to-face interactions emerged as the predominant offline method, with 90% (36/40) of the participants engaging with partners, 35% (14/40) with friends, 10% (4/40) with colleagues, and 10% (4/40) with extended family. Phone calls were used by 5% (2/40) of the participants when communicating with extended family. In the online realm, 65% (26/40) of the participants opted for online messaging apps with partners, 18% (7/40) with friends, 10% (4/40) with colleagues, 10% (4/40) with extended family, and 2% (1/40) with physicians or midwives. Online messaging app was used by 10% (4/40) of the participants with their friends and 20% (8/40) of the participants with their extended families. Notably, of the participants, the direct share feature on social media platforms was used by 35% (14/40) with partners, 5% (2/40) with friends, and 5% (2/40) with colleagues. The tag feature on social media was chosen by 10% (4/40) of the participants with partners and 5% (2/40) of the participants with friends. For broader dissemination, 5% (2/40) of the participants utilized the post feature, and 5% (2/40) of the participants engaged in the comment feature on social media platforms when targeting social media audiences.

**Table 2:** Information sharing strategies (N=40).

Communication channels and audience			Participants, n (%)
**Offline**			
	**Face-to-face**		
		Partner	36 (90)
		Friends	14 (35)
		Colleagues	4 (10)
		Extended family	4 (10)
		Physicians or midwives	14 (35)
	**Phone calls**		
		Extended family	2 (5)
**Online**			
	**Online messaging**		
		Partner	26 (65)
		Friends	7 (18)
		Colleagues	4 (10)
		Extended family	4 (10)
		Physicians or midwives	1 (2)
	**Online group messaging**		
		Friends	4 (10)
		Extended family	8 (20)
	**Direct share** **feature on social media**		
		Partner	14 (35)
		Friends	2 (5)
		Colleagues	2 (5)
	**Tag** **feature on social media**		
		Partner	4 (10)
		Friends	2 (5)
	**Post** **feature on social media**		
		Social media audiences	2 (5)
	**Comment** **feature on social media**		
		Social media audiences	2 (5)

#### The Reasons: Why Is Information Shared?

Table 3 presents 3 themes categorizing first-time fathers’ reasons for sharing information. These themes are reciprocity and validation of information, compassion and sympathy, and information enhancement. Participants’ reasons for sharing information mostly fit in the reciprocity and validation of information theme (14/40, 35%), followed by the compassion and sympathy theme (13/30, 32%). The least number of reasons fit under the information enhancement theme (9/40, 22%).

**Table 3:** Themes of reasons for sharing information about pregnancy (N=40).

Theme	Participants, n (%)	Quotes
Reciprocity and validation of information	14 (35)	“I share pregnancy information to initiate a discussion. I also hope to get advice from others based on the discussion results.” “One way to validate the information that I have is by sharing it with others and letting them give feedback on the information given. I can get another perspective by sharing the information obtained.” “During the casual hangout, I share information about pregnancy with my other friends. So in that way, I can compare the information that I have and make it more comprehensive.”
Compassion and sympathy	13 (32)	“I know how confused I was when I dealt with something related to pregnancy problems. So, if my friends facing difficulties or asking for advice, and I have the information, then I will share it with them.” “When I have good or interesting information and I consider it as valid information, I will share it with my partners and my friends so they can also benefit from it.” “Sometimes I share experiences of what I have done based on information obtained from the Internet to others who have similar problems. Or at least, they can take preventive actions based on my experiences.”
Information enhancement	9 (22)	“Sometimes I share information that I obtained with my partners or my families, so they can add or complete the information that I missed.” “What I have in mind when I share the information I obtained from the Internet with my partner is that she can give me a response whether she experiences or feels the same thing or maybe if she hasn’t and we need to figure it out further.”

In the reciprocity and validation of information theme, participants expressed their motivation to share pregnancy information as a means to initiate discussions and seek advice. Sharing information served as a method for validating the information they possess, obtaining feedback, and gaining additional perspectives. During face-to-face conversations, participants engaged in mutual sharing, fostering a collaborative environment where they compare and enhance their collective understanding of pregnancy-related information.

In the compassion and sympathy theme, participants highlighted their tendency to share information not only to support their partner but also when their friends face difficulties or seek advice related to pregnancy problems. The desire to assist others in navigating the challenges of pregnancy was evident, with participants sharing information with friends. In addition, participants found value in sharing personal experiences based on information obtained from the internet, aiming to provide insights and preventive actions for those facing similar issues.

In the information enhancement, participants mentioned sharing information either with their partners or others to complement or complete information they may have missed. Participants sought to enrich their understanding by incorporating the perspectives and insights of others.

#### The Reasons: Why Is Information Shared With Their Partner Only?

The exploration into why most first-time fathers share pregnancy information with their partners only resulted in thematic clusters, as outlined in Table 4. These findings encompass 5 key themes: asking over sharing (12/40, 30%), no relevant audience (5/40, 12%), self-situation awareness (5/40, 12%), privacy and intimacy (3/40, 8%), and physician-specific constraints (11/40, 28%).

**Table 4:** Reasons for sharing information about pregnancy only with their partner.

Theme	Participants, n (%)	Quotes
Asking over sharing	12 (30)	“Mostly, I prefer to ask others and the doctors rather than share first (even though it continues with asking or validating). By asking, after I get the answers, then I can just privately compare them with my information.” “During casual hangouts with friends, when we discuss pregnancy, I’d rather ask questions rather than share information. That is my preference, even though sometimes I already possess some of the information. I am afraid if I share incorrect information, they will judge me as an irresponsible expectant father.”
No relevant audience	5 (12)	“I used to share everything with my close friends. But now, they live far away, so it is difficult to share pregnancy information with them. We are not comfortable sharing this kind of information with colleagues or neighbours. Therefore, I only share information about pregnancy with my partner.” “We are the youngest couple in our circle both in friendship, in a work environment, and families. They already have children. No other couples with a similar situation. It’s unlikely for us to share pregnancy information.” “I don’t know where and to whom I share the information. As far as I know, there is no online forum for me (and other fathers) to share information about pregnancy. Maybe, I can comment on social media posts. But the other commenters are mothers, so I am not very comfortable with it (joining the conversation).”
Self-situation awareness	5 (12)	“I think I do not have relevant information to share. I feel that the others have more information than me. Thus, I don’t share any information with others.” “I feel like I am not an expert in this field. I only have a little knowledge about pregnancy. So, I am afraid that I might give false information to others that might harm them.” “This is the first time I have searched and obtained information about pregnancy. I am a first-time father. I don’t have any experience with this before. I need to have more experience before I can confidently share information with others.”
Privacy and intimacy	3 (8)	“I think information related to pregnancy, particularly if it is related to our partner’s condition, I consider it as private and intimate information. Thus, I don’t share it with others, other than my partner.” “Because we are not announcing pregnancy, I don’t share any pregnancy information with others. In my culture, it’s prohibited to announce pregnancy as it can heavily impact the family (particularly the mother) if there is something wrong with the baby. We will announce the baby’s birth, but not the pregnancy.”
Physician-specific constraints	11 (28)	“The doctor is very busy. He has so many patients. Our antenatal care meeting is very short and concise. It’s already filled with procedures for health checking. So, there is no time to have a longer conversation.” “My doctor is very famous but he is also a very strict guy. He doesn’t like to answer questions other than what is in the meeting session scope. We just do the antenatal care procedures and nothing else.” “I feel that we go to the doctors to get examined by the experts. They have been studying pregnancy for years. So, I trust them to give the valid information. I just silently compared to information obtained by myself. Furthermore, they have a high social status in society. I won’t bother them with questions from a person with a lack of literacy in pregnancy matters (me).” “The ob-gyn doctor already explains everything in detail (I think). Therefore, I rarely have further questions or need to validate any information obtained online.”

The first theme, “asking over sharing,” manifests as first-time fathers expressing a preference for asking others rather than actively sharing information. This behavior extends not only to interactions with physicians but also with friends or colleagues who have experienced pregnancy. First-time fathers articulated a preference for asking questions rather than potentially facing judgment by sharing information they obtained. In addition, first-time fathers stated that the feeling of being judged does not appear when sharing information with their partners.

The second theme, “no relevant audience,” emerges from first-time fathers living far away from the intended audience, particularly family and close friends, or perceiving their situation as unique. First-time fathers prefer face-to-face conversations for sharing information, and distance creates barriers in this regard. Some first-time fathers noted a lack of friends in similar pregnancy-related situations. This limits their willingness to share information with others.

The third theme, “self-situation awareness,” encapsulates first-time expectant fathers’ acknowledgment of their novice status in the field and lack of experience. This self-awareness influences their decision not to share information, reflecting thoughtful consideration of their knowledge and expertise. They recognize their status as beginners, feeling the need to gain more information, validate it, and accumulate personal experience before sharing. However, with their partners, first-time fathers are aware that their partners are also newcomers in this matter. Therefore, they can collaborate to improve their knowledge about pregnancy.

The fourth theme, “privacy and intimacy,” underscores first-time fathers’ perception of certain information as private. This emphasizes a desire to maintain confidentiality surrounding specific aspects of pregnancy. Furthermore, some cultural matters prevent them from announcing a pregnancy before certain weeks of pregnancy. This limits their opportunity to share pregnancy-related information with others. Therefore, first-time fathers keep the boundary by only sharing pregnancy-related information with their partners, especially related to their partner’s health situation.

Finally, “physician-specific constraints” form a distinctive theme encompassing various constraints related to physician-patient communications that are different from their communication constraints with others (ie, friends, colleagues, and extended family members). Factors such as limited meeting time, feeling unwelcome or encountering unresponsiveness, respecting physicians’ roles, and perceiving a communication gap influence fathers’ decisions not to share information in the medical context. These constraints create situations where fathers find themselves unable to share their findings on pregnancy information, leading to a passive role in discussions with health care experts. However, while addressing communication situations with their partners, first-time fathers did not encounter any of these limitations.

### Non–Information-Sharing Activities of First-Time Fathers

A small number of participants, specifically 10% (4/40) of the participants, did not share pregnancy-related information with anyone (Table 5). In total, 2 themes emerged; these themes were partners’ health qualifications (2/40, 5%) and work environment (2/40, 5%). Under the partner health qualification theme, the reason for not sharing information was because of the partner’s professional qualifications in the health care field (eg, physician or midwife). This led first-time fathers to perceive their partners as possessing superior knowledge about pregnancy. Consequently, they deemed it unnecessary to gain information about pregnancy intensively, including sharing any pregnancy-related information. Another factor influencing nonsharing was the demanding work environment of the respondent, such as military service, which was perceived as a hindrance to engaging in discussions about pregnancy. Furthermore, they lived apart from their partners daily, so they were not involved in their partners’ pregnancies. Despite not sharing information, these fathers still searched for pregnancy-related information, mainly driven by personal curiosity and preparation for the baby’s delivery.

**Table 5:** Reasons for not sharing information with anyone.

Theme	Participants, n (%)	Quotes
Partners’ health qualification	2 (5)	“My partner holds a degree in health/medicine. Therefore, I think I don’t need to find information intensively or share pregnancy-related information obtained as she knows better. I sometimes still search for information about pregnancy just in case, and also to prepare for the baby’s delivery and becoming a new parent.”
Work environment	2 (5)	“I work in the army and live afar with my pregnant partner and family. Both in my work and casual conversation environment, we rarely talk about our personal matters. Even though we talk about our personal topics, pregnancy is not included. However, in some break time, I occasionally search for information about pregnancy and parenting.”

## Discussion

### Summary of Key Findings

This study reveals that most (36/40, 90%) first-time Southeast Asian fathers share information with others. Most (22/40, 55%) first-time fathers share information only with their partners, while the remaining (14/40, 35%) extend this to a small circle, including physicians, extended family, and friends. The most popular communication method for sharing information is face-to-face (36/40, 90%), followed by online messaging apps (26/40, 65%). Social media sharing is less common, with 35% (14/40) of the participants engaging in private sharing and only 5% (2/40) sharing information publicly. First-time fathers’ motivations for sharing information include validating information (14/40, 35%) and compassion for others (13/40, 32%), with a smaller group aiming to enhance their understanding by adding others’ perspectives (9/40, 22%).

Among those who did not share information beyond their partners, the reasons included a preference for asking questions rather than sharing information (12/40, 30%), self-situation awareness (5/40, 12%), lack of relevant audiences (5/40, 12%), concerns about privacy (3/40, 8%), and physician-specific constraints (11/40, 28%). A small group of first-time fathers (4/40, 10%) did not share information with anyone, mostly due to their partner’s professional health qualification (2/40, 5%) or demanding work environments (2/40, 5%). However, these first-time fathers remained engaged in information seeking, driven by personal curiosity and preparation for the baby’s arrival.

### Sharing Approaches Depending on Audience

#### Overview

First-time fathers demonstrate various approaches to sharing information depending on the audience. This is influenced by the relational dynamic of the relationship with their audience [17,65]. In contrast to high-income countries where first-time fathers actively share pregnancy-related information on the internet publicly [11,23,26], in the Indonesian context, only a small number (2/40, 5%) of fathers post information about pregnancy on the internet publicly. This behavior stems from first-time fathers’ inclination to share pregnancy-related information predominantly with people they have formed close relationships with, such as their partners, close friends, or immediate family members [27].

Several factors may contribute to this observed difference in information-sharing behavior among first-time Southeast Asian fathers, particularly related to sharing information about pregnancy publicly. Cultural nuances, societal norms, and privacy concerns could be influential in shaping the information-sharing landscape in Indonesia [45]. The cultural context in Southeast Asia often places a high value on privacy, especially regarding personal matters, such as pregnancy [66]. This cultural emphasis on privacy may lead fathers to be more cautious about sharing such intimate details publicly, in contrast to the more open sharing practices observed in high-income countries. Moreover, the level of digital literacy and familiarity with online platforms may play a role. While there are many online sharing platforms in Southeast Asia, 10% (4/40) participants highlighted the lack of appropriate forums on social media for posting and engaging with other fathers to discuss pregnancy-related matters, leading to limited activity in social media environments concerning pregnancy. The findings also suggest a preference for face-to-face communication. Fathers in Indonesia may place a higher value on personal interactions and traditional modes of communication [41]. The findings suggest that first-time fathers feel more comfortable sharing information about pregnancy in more intimate and private situations and featuring 2-way communication. It reflects the importance of personal connections and the cultural context, where sharing sensitive information is often reserved for close-knit relationships in Southeast Asian societies [16].

Within this close-knit relationship, results of this study show that first-time fathers prioritize their partners as the primary audience for information sharing, with a substantial 90% (36/40) choosing to share pregnancy-related details. The strong inclination to share information with their partners reflects a foundational trust that underpins their communication, as first-time fathers feel secure when sharing sensitive information with their partners [17,18]. In addition, in this context, perceived rewards, such as increased emotional connection, mutual understanding, and the opportunity for shared decision-making, also encourage first-time fathers to share information [17]. This inclination also emphasizes the central role of the partner in the first-time expectant father’s information-sharing network [67]. The partner is considered a crucial and central figure in the information-sharing process. The partner’s role as the one carrying the child makes her a primary stakeholder in the pregnancy journey. First-time fathers might recognize that the well-being of the child and the success of the pregnancy are directly linked to the mother’s experiences and knowledge [65]. Sharing information with the partner may also contribute to her knowledge discovery. This information enhancement collaboration could help parents make better-informed decisions that might positively impact both the mother’s well-being and the neonatal development of the child [3]. The collaborative effort in seeking and sharing information may enhance the overall experience of pregnancy for both partners.

First-time fathers use various communication channels to share information about pregnancy with their partners. The importance of the information, types of information, and source of information may influence the choice of communication channel used [68]. Certain details or important information may be deemed more urgent or critical. Therefore, fathers might choose to use faster channels, such as online messaging. In addition, the nature of the information itself could dictate the preferred method of sharing. For instance, if there is a need to convey information with informative images, online methods might be favored. However, if first-time fathers need to share sensitive information, they may opt for face-to-face communication. In addition, first-time fathers may share information directly using social media features, as most of the information about pregnancy is found on social media. Thus, it might be more convenient for first-time fathers. However, in this reciprocity and validation of information, it remains unclear whether partners feel overwhelmed or have preferences regarding specific channels. Further investigation into partner preferences and perceptions of information-sharing methods would be valuable in understanding the dynamics of this communication process. This includes the frequency of receiving new information about pregnancy and the types of information.

While communicating with friends, fathers still assess the dynamics of the situation and the level of closeness with friends before sharing the information [20]. This assessment considers not only the potential benefits and risks for the first-time fathers but also the well-being and comfort of the friends as recipients of the information. Consequently, some first-time fathers express a preference for sharing sensitive pregnancy-related details in face-to-face interactions rather than through group communication channels. This inclination highlights the significance of trust in the sharing process, indicating that fathers feel the need to establish a level of trust with their friends before sharing or exchanging intimate information about the pregnancy [17,18]. This suggests that the nature of the information and the level of personal relationships play crucial roles in shaping first-time fathers’ choices of communication channels when sharing with friends.

With colleagues, most first-time fathers rarely share pregnancy-related information with them. This could be due to the desire to maintain a professional connection at work. However, first-time fathers express an intention to share information with their close colleagues, driven by a need for feedback from experienced fathers regarding pregnancy information they have acquired. Therefore, fathers emphasize that they are inclined to share pregnancy-related information primarily through face-to-face interactions, mostly during their lunch breaks. This practice may be influenced by the limited communication time fathers have with colleagues from the workplace. Moreover, first-time fathers choose to share information about pregnancy privately, specifically with their closest colleagues or those who are experiencing similar situations [17]. This result is consistent with the findings of the study by Widén and Hansen [27], emphasizing the importance of the interpersonal relationship with the audience, which often occurs through private conversations in sharing certain types of information.

Extended family also plays a role in the information-sharing network, with some participants relying on familial connections for sharing information about pregnancy. Most of these interactions occur in online group chat, and some of them use phone calls as they live separately with the other extended family members. Notably, first-time fathers express a willingness to share pregnancy-related information within the family, driven by the perceived expertise, experience, and credibility of certain members [17]. This willingness is particularly evident when family members possess relevant experiences in pregnancy or backgrounds in health sciences. First-time fathers aim to obtain responses and feedback when sharing pregnancy information to enhance the information they possess. Moreover, this tendency to share information to seek advice from older people resonates with findings in cultural studies conducted in Southeast Asia, as highlighted by Ford and Lyons [41]. This cultural aspect highlights the value placed on seeking guidance from more experienced individuals in the community. In addition, extended family members may recommend traditional methods or practices related to pregnancy that align with cultural and social norms in Southeast Asia [69]. Sharing pregnancy information with extended family in Southeast Asia goes beyond seeking advice; it is rooted in cultural and social norms that emphasize communal bonds and the importance of intergenerational wisdom [70].

Health care professionals, particularly physicians or midwives, were identified as pivotal figures in the information-sharing process, with 35% (14/40) of the participants expressing a preference for consulting medical experts. This choice could be motivated by the specialized knowledge and expertise that health care professionals bring to discussions [71]. Notably, it also reflects a cultural norm within Southeast Asian societies, where a high value is placed on the authority of medical professionals in matters related to health and childbirth [71]. The nature of information sharing with physicians or midwives occurred in the formal setting of one-on-one antenatal care meetings. In these limited-time interactions, first-time fathers engaged in sharing information with the implicit intention of initiating first-time further discussion or seeking validation of the information they have gathered [17]. However, the cultural context might play a pivotal role here, as the hierarchical structure in Southeast Asian societies underscores respectful communication with authority figures, particularly health care professionals. This could restrict physician-father communication, which aligns with the findings of the study by Claramita et al [72], suggesting that these interactions are confined within the formal boundaries of antenatal care settings. This nuanced approach reflects the broader cultural context in Southeast Asia, where respect for authority figures, particularly in the medical field, is ingrained in societal norms. This hierarchical gap might influence first-time fathers’ decisions to not share pregnancy-related information obtained with the physicians outside the antenatal care settings [72]. The study by Claramita et al [72] illuminates how patients face limitations in discussing or sharing any health information with their physicians due to various reasons, such as limited time, differences in social status, and communication gaps. Similar situations arise during antenatal care, providing fathers with a restricted opportunity to share the information obtained with health care professionals. When first-time fathers have an opportunity to interact with physicians, they tend to prefer asking questions rather than actively sharing information, a behavior often accompanied by seeking validation or clarification. This emphasizes the challenges and constraints within the health care setting that influence first-time fathers’ information-sharing practices.

On the basis of the various approaches discussed, technology, particularly online text messaging and social media platforms, plays an important role in shaping the information-sharing behaviors of first-time expectant fathers in Southeast Asia. These platforms offer accessible and convenient avenues for fathers to easily share the information found online [10]. First-time fathers use online messaging apps, such as WhatsApp, to share information obtained from website search results. In this case, the messaging platforms allow for direct, quick, and personal communication with their immediate circle. In addition to messaging platforms, first-time fathers optimize the use of social media’s sharing features when sharing information obtained with others. This feature allows first-time fathers to share posts, videos, and expert advice more efficiently [9]. However, when using this feature, most first-time fathers in Southeast Asia tend to opt for private sharing with others over public sharing on social media [30]. This may also explain why first-time fathers prefer Instagram and Facebook over Twitter (subsequently rebranded X), as Twitter is primarily used for public sharing rather than private sharing, which raises concerns about privacy [30]. This suggests that first-time fathers are leveraging these features for their convenience and efficiency rather than the capabilities to amplify and disseminate information to wider audiences. Consequently, first-time fathers might limit the opportunities to receive feedback, advice, or validation from others, potentially hindering their ability to enrich their information-seeking and sharing experience in an online environment [67].

#### Reasons Behind Information-Sharing Decisions

First-time fathers who actively share pregnancy-related information emphasize the reasons they share pregnancy information are to validate the information, be compassionate to others, and enhance the overall quality and completeness of pregnancy-related information. By participating in information-sharing activities, first-time fathers aim to initiate discussions, seek advice, exchange information for validation, and compare notes to enhance the comprehensiveness of the information they acquire. This finding aligns with the findings of studies by Savolainen [15] and Tangaraja et al [16], which underscores that individuals share information as a way to gather information through collaborative engagement. This reciprocity and validation of information among first-time fathers as new parents highlight the social nature of their information-sharing practices during the journey [73]. In addition, by sharing valuable and valid information and offering personal experiences for guidance, these fathers aim to contribute to the improvement of their relationship with other fathers [17]. The observed practices resonate with the broader social and cultural norms that emphasize the value of communal support and shared experiences in navigating significant life events [74]. This collaborative aspect of information sharing might have broader implications for paternal involvement in the pregnancy journey, as it fosters a sense of community and mutual support among fathers. Moreover, information enhancement motives highlight fathers’ active efforts to ensure the quality and completeness of shared knowledge. This underscores the importance of validation and cross-checking information before making decisions, which resonates with the results of a study by Fletcher and StGeorge [26], demonstrating that first-time fathers share information with the aim of bolstering their confidence. This study suggests that by receiving feedback and comments on shared information, first-time fathers might aim to enhance their understanding of pregnancy. Thus, it is expected to boost their confidence during the pregnancy journey. These findings suggest that the information-sharing practices of first-time fathers play a crucial role in fostering a supportive community and enhancing paternal involvement during the pregnancy journey.

Conversely, instances, where first-time fathers did not share information beyond their partners, reveal nuanced themes, including a preference for “asking” over “sharing,” a lack of intended audiences, self-situation awareness, privacy and intimacy, and physician-specific constraints. Most of the reasons for not sharing pregnancy information, as uncovered in this study, mirror the findings of a study by Onyeze-Joe and Godin [29], which was conducted in low- and middle-income countries. For instance, participants in both studies expressed reluctance to share pregnancy information due to their perceived self-awareness of unfamiliarity with the topics and a lack of experience. The cultural context emphasizing the privacy and intimacy of pregnancy information that leads first-time fathers to rarely share such experiences is also evident in the results of both studies. In addition, both studies highlight the impact of local cultural norms dictating that fathers keep pregnancy matters a secret and refrain from discussing them with others. Another concurrence is that first-time fathers do not share pregnancy matters due to concerns about being judged by other men [20]. Both studies revealed that first-time fathers, in light of potential judgment, prefer to ask questions rather than share information with others, particularly regarding new knowledge or experience. This suggests a shared cultural inclination influencing fathers’ information-sharing behaviors across multiple studies in low- and middle-income countries. These studies suggest that first-time fathers are reluctant to share pregnancy-related information beyond their partner due to cultural influence, situational constraints, and low capabilities.

These findings also indicate that support networks play an essential role in promoting information sharing and exchange during pregnancy [75,76]. Results of this study highlight that the primary themes behind the decision to share information—whether with others or with their partners only—heavily depend on the audiences who often act as support networks. For example, first-time fathers cite reasons for sharing information with others, such as reciprocity and validation of information, which underscores the importance of feedback from others as they seek affirmation to enhance their understanding. Furthermore, the most common reason for sharing with their partner only is “asking over sharing.” This means that first-time fathers need direct support from their partners in terms of obtaining pregnancy-related information. By enhancing support networks for first-time fathers, they can increase their confidence and improve their knowledge, which can lead to more involvement during pregnancy [75,76]. Furthermore, by creating intervention programs that encourage open discussions not only with partners but also within broader circles, including health care professionals and experienced extended family members, first-time fathers’ involvement in the pregnancy journey can be improved.

### Limitations and Future Directions

Several limitations should be acknowledged while interpreting the findings of this study. First, the study primarily relies on self-reported data from an interview, which introduces the possibility of social desirability bias and recall inaccuracies. In addition, the nature of the research design restricts the ability to capture the dynamic nature of information-sharing behaviors over time. Furthermore, the study’s focus on Southeast Asian cultural contexts may limit the applicability of the findings to other cultural settings.

Despite efforts to ensure diversity, the study’s geographical concentration in Indonesia might not fully capture the regional variations within the country. Finally, the rapidly evolving digital landscape introduces a temporal dimension to the study, and the information-sharing behaviors observed may evolve with technological advancements. These limitations should be considered when interpreting the findings, and they underscore the need for future research to address these constraints and further refine our understanding of first-time expectant fathers’ information-sharing practices.

A potential avenue for further exploration related to this topic is delving into the impact of cultural variations within low- and middle-income countries on fathers’ information-sharing behaviors during pregnancy. This could involve comparative studies across different regions or cultural subgroups within Southeast Asian countries, offering nuanced insights into how cultural diversity influences communication patterns. In addition, future research could investigate the role of digital literacy and access in shaping information-sharing practices among expectant fathers. Understanding how varying levels of digital literacy impact the use of online platforms for sharing pregnancy-related information could provide valuable insights for designing targeted interventions or support systems. Furthermore, longitudinal studies tracking fathers’ information-sharing behaviors throughout different phases of pregnancy and early parenthood could uncover how these practices evolve over time. Exploring how fathers’ roles and information-sharing dynamics shift from pregnancy to post partum could also contribute to a more comprehensive understanding of their evolving roles within the extended family unit. Finally, as demographic factors were not included in the analysis for this research study, exploring the relationship between sociodemographic aspects and information-sharing behavior could provide deeper insights into the dynamics of paternal involvement during pregnancy.

### Conclusions

In conclusion, the results of this study provide an understanding of the intricate interplay of factors that influence information-sharing practices among first-time expectant fathers in low- and middle-income countries. The existing gap in research on this subject prompted this study’s exploration, recognizing the unique cultural, digital, and contextual nuances within Indonesia. The country’s diversity in cultural norms and varying degrees of digital literacy serve as a rich backdrop for comprehending how fathers engage in the sharing of pregnancy-related information. By focusing specifically on Indonesia, with its diverse cultural landscape and regional commonalities, this study contributes essential insights that might be extrapolated to other Southeast Asian countries. In doing so, it addresses the existing research gap and enriches the global understanding of fathers’ roles in the intricate journey of pregnancy. This not only increases our knowledge of paternal engagement but also emphasizes the importance of nuanced approaches that take cultural variation into account in future theoretical discussions.

On a practical level, this study offers valuable insights with implications for policy makers, health care providers, and support organizations. On the basis of this study’s findings, physicians and midwives in Indonesia might need to allocate additional time and diversify communication channels during antenatal care to enhance paternal involvement. Developers can leverage these insights to create dedicated online forums tailored for fathers. For governmental intervention programs, the study suggests initiating awareness campaigns, workshops, and support groups to encourage fathers’ active participation in pregnancy discussions. In the workplace, employers can foster an inclusive environment through programs addressing the unique needs of expectant fathers, creating designated spaces for open discussions, and implementing awareness campaigns to destigmatize pregnancy conversations. These contributions aim to promote paternal involvement, enhance support networks, and foster a collaborative sharing environment. The extrapolation of findings to other Southeast Asian countries provides actionable knowledge for those working in the region that guides the development of culturally tailored strategies to promote paternal involvement during pregnancy. Overall, the study bridges the gap between theoretical advancements and practical applications in the context of paternal involvement in low- and middle-income countries.
